# Simultaneous Immunization with Multiple Diverse Immunogens Alters Development of Antigen-Specific Antibody-Mediated Immunity

**DOI:** 10.3390/vaccines9090964

**Published:** 2021-08-28

**Authors:** Kelsey A. Pilewski, Kevin J. Kramer, Ivelin S. Georgiev

**Affiliations:** 1 Vanderbilt Vaccine Center, Vanderbilt University Medical Center, Nashville, TN 37232, USA; kelsey.a.pilewski@vanderbilt.edu (K.A.P.); kevin.j.kramer@vanderbilt.edu (K.J.K.); 2 Department of Pathology, Microbiology, and Immunology, Vanderbilt University Medical Center, Nashville, TN 37232, USA; 3 Vanderbilt Institute for Infection, Immunology, and Inflammation, Vanderbilt University Medical Center, Nashville, TN 37232, USA; 4 Department of Electrical Engineering and Computer Science, Vanderbilt University Medical Center, Nashville, TN 37232, USA; 5 Program in Computational Microbiology and Immunology, Vanderbilt University Medical Center, Nashville, TN 37232, USA

**Keywords:** immunization, antibodies, subunit vaccines, humoral immunity, bacterial pathogens

## Abstract

Vaccination remains one of the most successful medical interventions in history, significantly decreasing morbidity and mortality associated with, or even eradicating, numerous infectious diseases. Although traditional immunization strategies have recently proven insufficient in the face of many highly mutable and emerging pathogens, modern strategies aim to rationally engineer a single antigen or cocktail of antigens to generate a focused, protective immune response. However, the effect of cocktail vaccination (simultaneous immunization with multiple immunogens) on the antibody response to each individual antigen within the combination, remains largely unstudied. To investigate whether immunization with a cocktail of diverse antigens would result in decreased antibody titer against each unique antigen in the cocktail compared to immunization with each antigen alone, we immunized mice with surface proteins from uropathogenic *Escherichia coli*, *Mycobacterium tuberculosis*, and *Neisseria meningitides*, and monitored the development of antigen-specific IgG antibody responses. We found that antigen-specific endpoint antibody titers were comparable across immunization groups by study conclusion (day 70). Further, we discovered that although cocktail-immunized mice initially elicited more robust antibody responses, the rate of titer development decreases significantly over time compared to single antigen-immunized mice. Investigating the basic properties that govern the development of antigen-specific antibody responses will help inform the design of future combination immunization regimens.

## 1. Introduction

The design and implementation of successful immunization regimens worldwide have cemented vaccination as one of the most important human medical interventions in history [1,2,3,4,5]. However, traditional vaccination strategies utilizing immunization with a live-attenuated or inactivated agent have proven insufficient in the face of many contemporary epidemic, highly mutable, and emerging pathogens [6,7]. By contrast, modern strategies aim to rationally engineer a single antigen or cocktail of antigens to generate a more focused, protective immune response [8,9,10,11]. Further, although vaccine platform and formulation have been shown to have a profound effect on the magnitude and quality of the elicited immune response [9,12,13,14], the effect cocktail vaccination (simultaneous immunization with multiple immunogens) has on the antibody response to each individual antigen within the combination remains largely unstudied.

In this study, we sought to characterize the effect of cocktail vaccination on the immunogenicity of pathogen-derived protein antigens. We hypothesized that immunization with a cocktail of structurally, and functionally diverse antigens would result in decreased antibody titer against each unique antigen in the cocktail, compared to mice immunized with each antigen alone. To investigate this, we immunized mice with cell surface-exposed proteins from uropathogenic *Escherichia coli*, *Mycobacterium tuberculosis*, and *Neisseria meningitides*, and monitored the development of antigen-specific IgG antibody responses in BALB/c mice. 

## 2. Materials and Methods

### 2.1. Antigen Expression and Purification

The gene encoding the bacterial antigen HBHA (GenBank: AAC26052.1) was synthesized by Genscript (Genscript, NJ, USA) and cloned in the pET9a bacterial expression vector. The gene encoding a rationally designed fHbp construct with a 6X HisTag was synthesized by Genscript and cloned in the pET9a bacterial expression vector [15]. The pET30b+ plasmid containing the gene for HisTagged-IreA was a gift from Harry T. Mobley (University of Michigan) [16]. 

For protein expression, plasmids described above were transformed into the appropriate *E. coli* strains and cultured in Luria Broth with 50 μg/mL Kanamycin (+25 μg/mL chloramphenicol for BL21 (DE3) pLysS cell culture). Recombinant protein expression from pET9a was induced in Rosetta (DE3) cells at OD_600_ = 0.6 with the addition of 1 M isopropyl β-D-1-thiogalactopyranoside (IPTG) (added to a final concentration of 1 mM) for 6 h at 37 °C with shaking. Recombinant protein expression from pET30b+ was induced in BL21 (DE3) pLysS cells at OD_600_ = 0.8 with the addition of 1 M isopropyl β-D-1-thiogalactopyranoside (IPTG) (added to a final concentration of 1 mM) for 6 h at 37 °C with shaking. 

Induced cultures were harvested by centrifugation (8000× *g*, 4 °C, 20 min) and pellets frozen at −80 °C overnight. Pellets were then thawed on ice and resuspended in 5 mL/g pellet weight with the appropriate binding buffer (+EDTA-free protease-inhibitor (Roche)) before lysis by 6 × 30 s rounds of sonication. Lysate was cleared by centrifugation (10,000× *g*, 4 °C, 20 min) and filtered using a 0.22 μm PES filter before protein purification. 

Purification of HBHA: Pellets containing HBHA expressed from pET9a were resuspended in binding buffer (10 mM sodium phosphate, pH = 7) and lysate prepared as described above. HBHA was purified by multiple rounds of heparin affinity purification using an equilibrated 5 mL pre-packed Heparin HiTrap HP column (GE Healthcare, IL, USA). The column was washed with 10 column volumes (CV) of binding buffer, and purified protein was eluted from the column with a 25 mL gradient into binding buffer +2 M NaCl, pH = 7. 

Purification of fHbp: Pellets containing fHbp expressed from pET9a were resuspended in binding buffer (20 mM sodium phosphate, 0.5 M NaCl, 10 mM imidazole, pH 7.4) and lysate prepared as described above. fHbp was purified by nickel affinity chromatography using an equilibrated, 5 mL pre-packed HisTrap HP column (GE Healthcare, IL, USA). The column was washed with 10 CV of binding buffer, and purified protein was eluted from the column with a 25 mL gradient into binding buffer +0.5 M Imidazole, pH = 7.4.

Purification of IreA: Pellets containing IreA expressed from pET30b+ were resuspended in denaturing binding buffer (20 mM Tris-Cl, 0.5 M NaCl, 10 mM imidazole, 6 M guanidine-HCl, 1 mM β-mercaptoethanol, 1% Triton X-100, pH 8), and allowed to incubate with stirring for 1 h, before clearing the lysate as described above. IreA was purified using an equilibrated, 5 mL pre-packed HisTrap HP column (GE Healthcare, IL, USA). The column was washed with 10 CV wash buffer (20 mM Tris-Cl, 0.5 M NaCl, 10 mM imidazole, 6 M Urea, 1 mM β-mercaptoethanol, 1% Triton X-100, pH = 8), before on-column protein re-folding using a 50 mL gradient into renaturation buffer (20 mM Tris-Cl, 0.5 M NaCl, 10 mM imidazole, 1 mM BME, 1% Triton X-100, pH = 8). Finally, purified protein was eluted from the column with a 25 mL gradient into 20 mM Tris-Cl, 0.5 M NaCl, 0.5 M imidazole, 1 mM BME, 0.05% Triton X-100, pH = 8.

All purified recombinant proteins were buffer-exchanged 5X into sterile phosphate-buffered saline (PBS), and their concentrations determined by BCA assay (Pierce, MA, USA).

### 2.2. Vaccination

Six- to eight-week-old female BALB/c mice (*n* = 5/group) were used for these studies, and animals were ≤15 weeks old at study conclusion. All procedures were conducted according to protocols approved by Institutional Animal Care and Use Committee at Vanderbilt University Medical Center.

Purified protein combinations were diluted in sterile PBS and emulsified 1:1 in TiterMax Gold (Sigma-Aldrich, MO, USA) for intraperitoneal injection. Isoflurane-anaesthetized mice were immunized on day 0 and received booster injections on days 28 and 56 with either 267 pmol of each antigen alone, each combination of two antigens, or all three antigens according to vaccination group (see Table 1 below). Blood was collected 14 days after each immunization (days 14, 42, and 70) by submandibular puncture. At study conclusion, mice were sacrificed by CO_2_ overdose and cardiac puncture exsanguination. Blood was allowed to clot at room temperature and serum separated by centrifugation (10,000× *g*, 4 °C, 10 min). Serum was transferred to a new tube and stored at −80 °C until use. 

### 2.3. Enzyme-Linked Immunosorbent Assay (ELISA)

For indirect serum Enzyme-linked Immunosorbent Assay (ELISA), Immulon 2HB plates (Nunc) were coated with 2 μg/mL of purified recombinant antigen diluted in PBS overnight at 4 °C. Excess antigen was removed with 3X wash with PBS+ 0.05% Tween-20 (PBS-T). This washing step was repeated after each subsequent incubation step. Non-specific binding was blocked with 5% non-fat dried milk (NFDM) in PBS for 1 h at 37 °C, followed by washing. Hyperimmune sera was serially diluted in 1% NFDM in PBS-T, added to wells, and incubated for 1 h at 37 °C. Plates were washed, and incubated with anti-mouse IgG-HRP diluted 1:10,000 in 1% NFDM in PBS-T for 1 h at 37 °C. After washing, plates were developed with 3,3′,5,5′-Tetramethylbenzidine for 10 min in the dark, reaction stopped with 1N sulfuric acid, and absorbance read at 450 nm. All ELISA data shown and used for calculations was blank subtracted. Endpoint dilution titer was defined as the serum dilution at which binding reached the lower limit of detection (OD_450_ = 0.1). 

### 2.4. Statistical Analysis

All graphing and statistical analyses were done using GraphPad Prism 9. Significance between all immunization groups was determined using Kruskal–Wallis test (with Dunn’s test for multiple comparisons). Significance between pairwise combinations of immunization groups was determined by Mann–Whitney U test. ELISA antibody endpoint titers were determined by interpolating a standard curve using GraphPad Prism 9. Half maximal effective concentrations (EC_50_) were determined by interpolating a standard curve using GraphPad Prism 9. All statistics were conducted using 95% confidence intervals where applicable.

## 3. Results

### 3.1. Simultaneous Immunization with Multiple Diverse Immunogens

**Figure 1:**
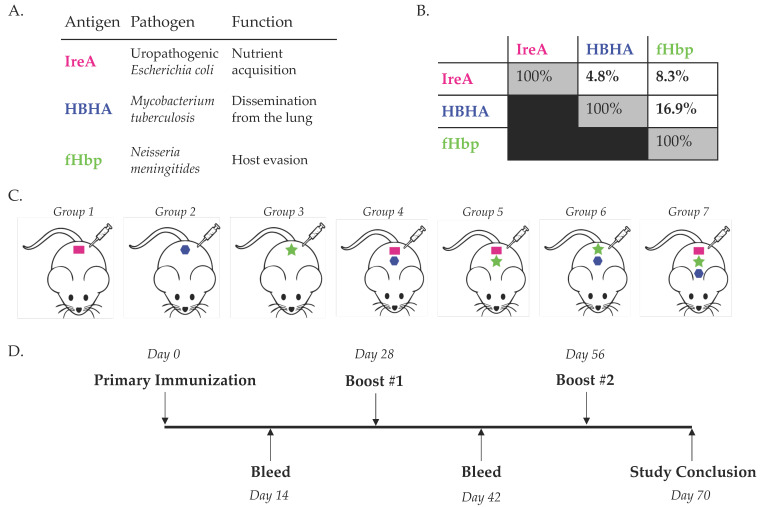
**Study Design for Simultaneous Immunization with Diverse Antigens**; (**A**) Table of antigens used for immunization in this study. Each of these antigens has been tested at least pre-clinically as a vaccine candidate against their respective native host [15,16,20]. (**B**) Percent sequence identity overlap between each of the immunogens utilized. (**C**) Immunization schedule and antigen groups for this study denoted by colored symbols (Pink: IreA, Blue: HBHA, Green: fHbp). *n* = 5 female BALB/c mice/group. (**D**) Immunization and bleed regimens used for all groups.

To investigate the effect of cocktail (vs. single antigen) immunization on the development of humoral immunity, we immunized BALB/c mice (*n* = 5/group) with equimolar quantities of either three diverse immunogens, each unique combination of two, or each immunogen alone, and monitored the development of antigen-specific IgG antibodies. We selected three functionally diverse antigens from divergent pathogens that had either previously been tested or approved as vaccination targets against their native hosts after eliciting protective antibody responses in mice. Specifically, we chose the iron-regulated outer membrane protein IreA, from uropathogenic *Escherichia coli*, which is exclusively expressed by pathogenic strains of *E. coli* and facilitates nutrient metal acquisition [16,17,18]; the heparin-binding hemagglutinin protein HBHA from *Mycobacterium tuberculosis*, which facilities bacterial dissemination from the lung [19,20,21]; and the factor H-binding protein fHbp from *Neisseria meningitides*, which facilitates bacterial innate immune evasion [15,22,23] (Figure 1A). Consistent with their divergent cellular functions, these proteins share low sequence identity (Figure 1B). We immunized mice with each combination of antigens described above followed by two booster immunizations, and collected serum 14 days after each immunization for serological analysis (Figure 1C,D).

### 3.2. Antigen Immunization Combinations Elicit Comparable Antibody Titers by Study Conclusion

**Figure 2:**
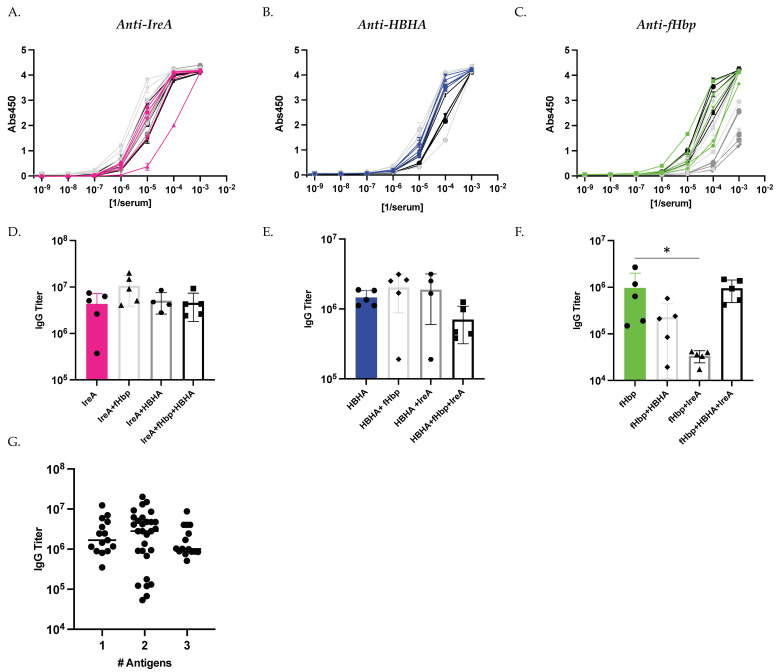
**Antigen Immunization Combinations Elicit Comparable Antibody Titers by Study Conclusion**. Antigen-specific IgG serum antibody titer measured at study conclusion. Endpoint antibody titer against IreA (**A**), HBHA (**C**), and fHbp (**E**) was measured by direct ELISA using serum from each immunization group shown. Quantification of endpoint titers for each immunization group against IreA (**B**), HBHA (**D**), and fHbp (**F**). Colored lines, and associated bar graphs, represent responses in single antigen immunized mice (IreA, pink; HBHA, blue; fHbp, green). Black lines, and associated bar graphs, represent responses in cocktail (triple antigen)-immunized mice. Gray lines represent mice immunized with each combination of two antigens, each group is denoted by a different symbol. (**G**) Comparison of antigen-specific antibody titers and number (#) of antigens with which a mouse was immunized. Statistical significance was determined by Kruskal-Wallis test. * denotes *p* < 0.05.

After completion of the described vaccination regimens with diverse antigen combinations, we sought to compare the elicitation of antigen-specific IgG antibody responses between each immunization group. We measured the serological antibody response to each individual immunogen using ELISA (Figure 2A–C) and quantified antigen-specific antibody titers (Figure 2D–F). We observed that IgG titers elicited against both IreA and HBHA were comparable by the end of the study regardless of vaccination group (Figure 2A,B,D,E). By contrast, responses to fHbp showed more variability between groups, and mice immunized with IreA+fHbp displayed significantly decreased fHbp-specific anti body titers compared to mice immunized with fHbp alone (Figure 2C,F). Overall, we did not observe a relationship between the number of antigens with which each mouse was immunized and the magnitude of the elicited antibody response, with no statistically significant correlation between immunization groups with different numbers of antigens (Figure 2G). 

### 3.3. Vaccination Type Affects Development of Antigen-Specific Antibody Titers

**Figure 3:**
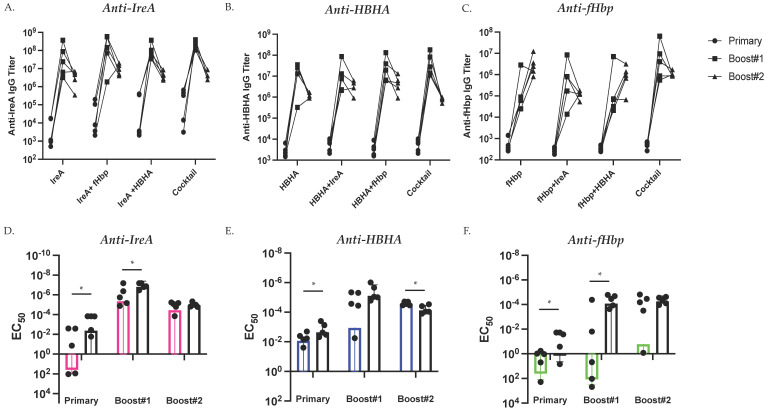
Cocktail Immunization Initially Elicits Higher Antibody Titers. Development of antigen-specific serum IgG antibody titers against IreA (**A**), HBHA (**B**), and fHbp (**C**) by each immunization group. Comparison of endpoint titer between single antigen (colored line)- or triple antigen (black line)-immunized mice determined by ELISA 14 days after primary immunization (left), boost #1 (middle), and boost #2 (right). (**D**) The EC_50_ (concentration of serum at which the half-maximal response is observed) against IreA is quantified over time for both IreA-immunized (pink line) and HBHA+fHbp+IreA-immunized (black lines). (**E**) The EC_50_ against HBHA is quantified over time for both HBHA-immunized (blue lines) and HBHA+fHbp+IreA-immunized (black lines). (**F**) The EC_50_ against fHbp is quantified over time for both fHbp-immunized (green line) and HBHA+fHbp+IreA-immunized (black lines). Statistical significance was determined by Mann-Whitney U test. * denotes *p* < 0.05.

After discovering comparable antigen-specific endpoint antibody titers against each individual immunogen across vaccination groups, we next sought to examine the development of this response across over time. We investigated the effect of vaccination type (single subunit vs. cocktail) on the serological antibody response to each antigen ~14 days after each immunization (Figure 3). We measured serum antibody responses against each individual immunogen using ELISA and quantified the antigen-specific antibody response elicited to each vaccination group over time (Figure 3A–C). The majority of mice immunized with IreA and HBHA displayed peak titer responses at day 42 (after boost #1) regardless of vaccination group (Figure 3A,B). Comparatively, we observed greater fluctuations in the fHbp-specific response between groups over time, with many mice achieving peak titers at day 70 (Figure 3C). We next compared the antigen-specific antibody titers elicited by mice immunized with a single antigen or all three antigens (cocktail-immunized) over time (Figure 3D–F). Interestingly, after primary vaccination, cocktail-immunized mice elicited more robust antibody responses against all three antigens (Figure 3D–F). Further, after boost #1 cocktail-immunized mice elicited more robust antibody responses against both IreA and fHbp, and although not statistically significant, anti-HBHA responses followed a similar trend (Figure 3D–F). In summary, we discovered that although antigen-specific antibody titers were comparable by study conclusion, cocktail immunization initially elicited more robust antibody titers than single antigen-immun ized mice.

### 3.4. Cocktail Immunization Alters Development of Antigen-Specific Antibody-Mediated Immunity 

**Figure 4:**
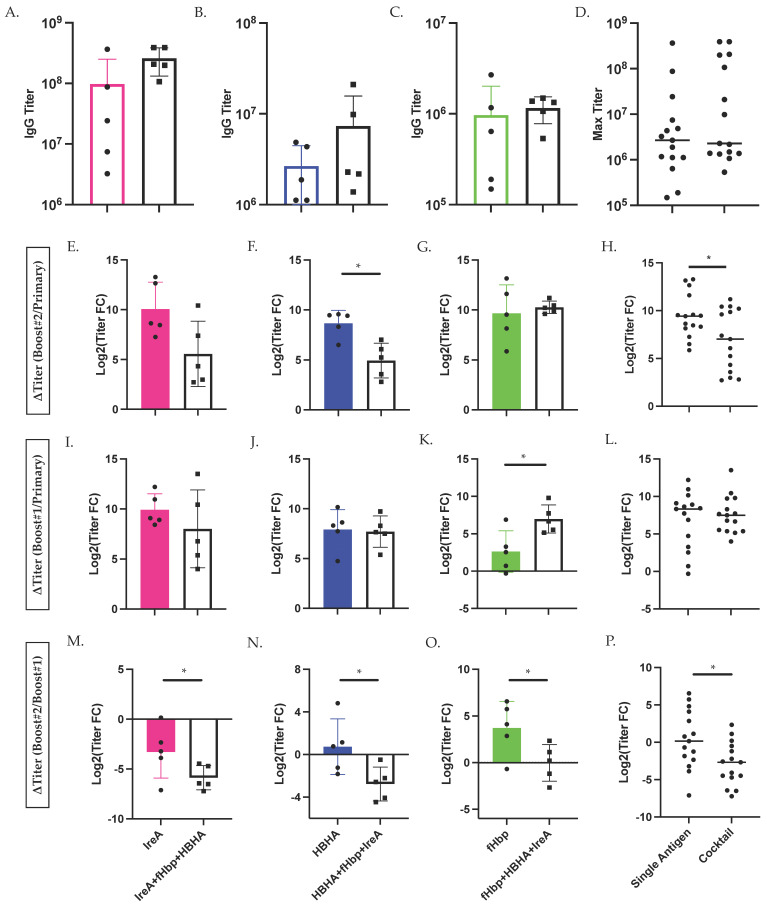
Cocktail Immunization Alters Development of Antigen-Specific Antibody-Mediated Immunity. Max endpoint serum antibody titer against IreA (**A**), HBHA (**B**), and fHbp (**C**) measured by ELISA in either single antigen- (color) or cocktail-immunized (IreA+fHbp+HBHA; black). Max serum antibody titer observed in all single antigen vs. cocktail-immunized mice (**D**). Change in serum antibody titers over the course of vaccination. Change in antibody titer between primary immunization and boost #1 (**E**), between boost #1 and boost #2 (**I**), and between primary immunization and boost #2 (**M**) in IreA- vs. IreA+fHbp+HBHA-immunized mice. (**F**,**J**,**N**) As described for IreA, observed changes in HBHA-specific antibody titers. (**G**,**K**,**O**) As described for HBHA, observed changes in fHbp-specific antibody titers. Change in antibody titer between primary immunization and boost #1 (**H**), between boost #1 and boost #2 (**L**), and between primary immunization and boost #2 (**P**) in all single antigen- vs. cocktail-immunized mice. Statistical significance was determined by Mann-Whitney U test. * denotes *p* < 0.05.

Although we found that antigen-specific antibody titer was not influenced by vaccination group at study conclusion, we discovered that cocktail-immunized mice initially elicited more robust serological antibody responses. In order to evaluate endpoint titer independent of timepoint, we compared the maximum antibody titer reached by mice immunized with either a single antigen or cocktail of antigens. We observed similar maximum titers across single- and cocktail-immunized mice (Figure 4A–D). We also compared the rate of antigen-specific IgG antibody development (change in titer over time) between single antigen and cocktail-immunized mice (Figure 4E–P). When we considered the change in titer between primary immunization and boost #2 as a function of antigen specificity, we observed an increased rate of titer development against both IreA and HBHA, although there was no difference in anti-fHbp responses (Figure 4E–G). We next combined these data to evaluate the change in titer as a function of immunization group, and discovered that over the length of the study, mice immunized with a single antigen showed an increased rate of antigen-specific IgG antibody titer over cocktail (triple antigen)-immunized mice (Figure 4H). When comparing antigen-specific antibody titer development between primary vaccination and boost #1, we observed no differences between immunization groups (Figure 4I–L). Finally, between boost #1 and the study conclusion, we found that cocktail-immunized mice showed a significant decrease in antibody titer change compared to single antigen-immunized mice (Figure 4M–P). Taken together, these findings suggest that cocktail immunization initially elicited more robust antibody responses but that the change in antibody titer development of these responses tapers more quickly over time. 

## 4. Discussion

Vaccines represent one of the most successful medical interventions in history, and their efficacy is dependent on the induction of a robust and long-lasting immune response, traditionally through the elicitation of neutralizing antibodies [24,25]. Modern vaccinology strategies are often focused on the rational design of a single antigen or a cocktail of antigens to generate a more focused, protective immune response [26,27,28,29]. In this study, we examined how simultaneous immunization with multiple diverse antigens affects the development of antigen-specific IgG antibody responses using a prime/two boost vaccination regimen in mice as a model. We discovered that primary immunization followed by two booster immunizations with different combinations of up to three soluble bacterial antigens elicited comparable endpoint antibody titers by study conclusion (day 70).

However, after prime and boost #1, mice vaccinated with all three antigens elicited significantly higher antigen-specific IgG antibody responses than mice immunized with a single antigen, while double antigen-immunized mice displayed an intermediate phenotype. When we compared the fold change in antigen-specific IgG antibodies over the course of the study, we found that single antigen-immunized mice showed an increased rate of antibody development over triple antigen-immunized mice. Finally, we observed that this difference could largely be traced to a significant decrease in antibody titers between booster immunizations #1 and #2 in triple antigen-immunized compared to single antigen-immunized mice.

Our observations described here, along with previous studies, suggest that cocktail administration of subunit immunogens alters the development of antigen-specific antibody responses [30]. Specifically, our results imply that immunization with multiple diverse antigens provides an initial boost to the immune response, and this strategy could be used to quickly elicit high IgG antibody titers against several immunogens. Multiple processes could explain these findings, including that immune exposure to increased antigenic diversity, in this case via cocktail immunization, recruits a greater heterogeneity of immune cells, leading to the formation of more robust germinal center reactions and class-switched antibody responses. By contrast, it could be that cocktail immunization elicits more cross-reactive and polyreactive antibodies, engendering higher apparent titers. However, to elucidate the mechanisms behind our observations, it will be important for future studies to investigate markers of immune activation, induction of memory cells and hallmark cytokines. Moreover, what effect this has on long-term immunological memory and recall in the context of human vaccination will need to be further examined.

Finally, we note several limitations to our study that may have influenced our observations, including the usage of all female mice, one mouse strain (BALB/c), and inconsistent total immunization mass between groups. This investigation was designed such that each mouse group received the same quantity of each unique antigen, although this means that cocktail-immunized mice received the greatest total antigen mass, which could indeed account for the high antibody titers elicited by this group. Nonetheless, we discovered that mice in groups 5 and 6 (IreA+fHbp, HBHA+fHbp) elicited lower endpoint antibody titers against fHbp than mice immunized with fHbp alone, despite being vaccinated with a larger total protein mass. These data suggest that our results cannot solely be explained by total vaccine antigen mass and could indeed be influenced by immunodominance or antigen-specific factors.

## 5. Conclusions

In summary, we observed that immunization with multiple diverse bacterial antigens initially induces more robust IgG antibody responses, but that this response wanes more quickly over time, compared with single antigen-immunized mice. Investigating the effect of antigenic properties and formulations on vaccination response, such as those described in this study, contributes both to our basic understanding of factors governing the development of antigen-specific immunity, as well as serves to inform future immunization regimens.

## Figures and Tables

**Table 1:** Total Immunization Mass Varies Between Groups.

Antigen	Group	1	2	3	4	5	6	7
IreA		20			20	20		20
HBHA			5.9		5.9		5.9	5.9
fHbp				7.4		7.4	7.4	7.4
	Total (μg)	20	5.9	7.4	25.9	27.4	13.3	33.3

Mass of each antigen utilized for immunization across groups such that ~267pmol of each unique antigen is administered per relevant group. Total immunization mass differs across groups.
